# Nordic Walking Promoted Weight Loss in Overweight and Obese People: A Systematic Review for Future Exercise Prescription

**DOI:** 10.3390/jfmk4020036

**Published:** 2019-06-18

**Authors:** Stefano Gobbo, Valentina Bullo, Enrico Roma, Federica Duregon, Danilo Sales Bocalini, Roberta Luksevicius Rica, Andrea Di Blasio, Lucia Cugusi, Barbara Vendramin, Manuele Bergamo, David Cruz-Diaz, Cristine Lima Alberton, Andrea Ermolao, Marco Bergamin

**Affiliations:** 1 Sport and Exercise Medicine Division, Department of Medicine, University of Padova, Via Giustiniani, 2, 35128 Padova, Italy; 2 Laboratorio de Fisiologia e Bioquimica Experimental, Centro de Educacao Fisica e Deportos, Universidade Federal do Espirito Santo (UFES), Vitoria, ES, Rua Vergueiro, 235, Liberdade, Sao Paulo, SP 01504-00, Brazil; 3 Departamento de Educacao Fisica e Ciencias do Envelhecimento, Laboratorio de Percepcao Corporal e Movimento, Universidade Sao Judas Tadeu, Sao Paulo, SP 05503-001, Brazil; 4 Department of Medicine and Sciences of Aging, G. d’Annunzio University of Chieti-Pescara IT, 100, 66100 Chieti, Italy; 5 Department of Medical Sciences and Public Health, University of Cagliari IT, SS 554, 09042 Monserrato, Italy; 6 Department of Health Sciences, Faculty of Health Sciences, University of Jaén, 23001, 23009 Jaén, Spain; 7 Physical Education School, Federal University of Pelotas, Rua Luís de Camões, Pelotas, RS 96055630, Brazil

**Keywords:** nordic walking, obesity, review, exercise prescription, weight loss

## Abstract

The aim of this systematic review was to analyze the effect of Nordic Walking (NW) on anthropometric parameters, body composition, cardiovascular parameters, aerobic capacity, blood sample, and glucose tolerance in overweight and obese subjects. The main keywords “Nordic Walking” or “Pole Walking”, associated with either “obese”, “obesity”, “overweight”, or “weight loss” were used on the online database MEDLINE, PubMed, SPORTDiscus and Scopus. Additionally, references of the studies included were screened to identify eligible articles. Applying the inclusion and exclusion criteria, ten manuscripts were considered as eligible for this review. The results of the studies were categorized in several domains with regard to “anthropometric parameters and body composition”, “cardiovascular parameters and aerobic capacity”, and “blood sample and glucose tolerance”. The results showed positive effects on the anthropometric parameters, body composition, cardiovascular parameters, blood sample, and glucose tolerance. The greatest improvements were observed in supervised and high weekly frequency of NW interventions. NW could be considered as an effective modality through which to involve the obese in physical activity. For weight loss, NW should be prescribed 4–5 times per week, at least 60 min per session, preferably combined with diet control.

## 1. Introduction

Physical inactivity is the primary cause of most chronic diseases and is responsible for accelerating biological aging becoming one of the risk factors for premature death worldwide [1]. Insufficient level of PA predispose people to an increased risk for developing chronic conditions, including diabetes, cardiovascular disease, and cancer [2]. Moreover, the abundance of energy-enriched food associated with a sedentary lifestyle has caused an increase in the number of overweight and obese people worldwide, including in developing countries [3]. The American College of Sport Medicine 2018 Guidelines recommendation for overweight and obese subjects suggest a dose-response relationship for weight loss. Namely, less than 150 min of weekly PA induces a minimal weight loss, more than 150 min induce a moderate weight loss, while 225–420 min of weekly PA results in a large weight loss. To promote long-term weight loss, PA should be performed 5–7 days per week for at least 250 min of weekly PA [4]. Compared to sedentary people, any level of leisure-time PA reduces the risk of mortality [5], also in the overweight and obese population [6]. Moreover, people who were physically active were less likely to gain weight [7], and the less active people had the greatest benefits from increasing their level of PA [8].

Walking is the most common modality to increase PA in sedentary people. Despite this, in young and healthy subjects, walking alone is not enough to reach the intensity necessary to induce training modification. Furthermore, an increase in walking speed should be required to reach the optimal training intensity. Unfortunately, in some morbid population such as people with obesity, that walking speed is difficult to be reached [9]. For this reason, Nordic Walking (NW) could be considered as an alternative method to increase exercise intensity and energy expenditure, especially for people with mobility impairments [9]. NW is a combination of walking and cross-country skiing performed with poles that are specifically developed for this activity. The involvement of the upper and lower limb, simultaneously induces an increase in energy expenditure, during NW practice, in comparison to normal walking [10]. The use of Nordic poles facilitates the practice in people with mobility impairments, like the elderly [11] and Parkinson disease patients [12]. In comparison with simple walking, the poles reduce the articular load on the lower limb during NW, preventing damage and pain to the knee joint, with potential benefits for people with obesity [13].

In light of these perspectives, the aim of this systematic review was to analyze the effect of NW programs on anthropometric parameters, body composition, cardiovascular parameters, aerobic capacity, blood sample, and glucose tolerance in overweight and obese subjects. Awaiting future and more solid evidences, the final goal of this review was to recommend an initial scheme to introduce NW in the exercise prescription for overweight and obese individuals.

## 2. Materials and Methods 

### 2.1. Study Design

This systematic review of the literature aimed to analyze the effects of NW on anthropometric parameters, body composition, cardiovascular parameters, aerobic capacity, blood sample, and glucose tolerance on overweight and obese subjects. The Preferred Reporting Items for Systematic Reviews and Meta-Analyses (PRISMA) guidelines and flow chart diagram were used as a reporting structure for this systematic review [14,15].

### 2.2. Literature Search

The literature research was performed between June and July 2018. The main keywords “Nordic Walking” or “Pole Walking”, and “obese”, “obesity”, “overweight”, or “weight loss” were used on the online database MEDLINE, PubMed, SPORTDiscus, and Scopus. In addition, references of the included studies were screened to identify the eligible articles.

### 2.3. Inclusion and Exclusion Criteria

Only studies published in English on peer-reviewed journals were considered for the inclusion. To be included, articles had to meet the following inclusion criteria, according to PICO model [15]—(a) Body Mass Index (BMI) higher than 25; (b) supervised or not supervised NW intervention; (c) the presence or not of a control group; (d) evaluation of anthropometric parameters, body composition, cardiovascular parameters, aerobic capacity, blood sample, and glucose tolerance; and (e) randomized controlled trial (RCTs) and no-randomized controlled trial (noRCTs). Both males and females from all races and different states of health were included. All studies that did not evaluate the outcomes through pre- and post-intervention comparisons, with mixed activity intervention, as well as cross-sectional studies and case reports, were excluded. Published abstracts, dissertation materials, or conference presentations were not considered as eligible documents.

### 2.4. Study Quality Assessment

The quality of the studies was assessed by applying an adapted nine criteria checklist, provided by the Cochrane Collaboration Back Review Group [16]. As in the previous review [17,18], the checklist had to be marginally adapted to rate the strength of the evidence. Each study in the review was scored on the basis of the following nine criteria: (1) “Was the method of randomization adequate?”; (2) “Were the groups similar at baseline regarding the outcome measures?”; (3) “Were inclusion and exclusion criteria adequately specified?”; (4) “Was the drop-out rata described adequately?”; (5) “Were all randomized participants analyzed in the group to which they were allocated?”; (6) “Was compliance reported for all groups?”; (7) “Was intention-to-treat analysis performed?”; (8) “Was the timing of outcomes assessment similar in all groups?”; and (9) “Was a followed up performed?”. When the paper provided a satisfactory description, a positive value was assigned (+). If the criterion descriptions was considered absent, unclear, or lacked the specified content, a negative value was assigned (−). A study was qualitatively estimated as high quality, if it showed a positive score on 5 out of 9 of the criteria; otherwise, it was considered a low-quality study.

### 2.5. Data Extraction and Synthesis

Two researchers independently examined all abstracts of the sourced studies. Several studies were analyzed in more details to be included in the review. Additional articles were sourced, reviewing the reference sections of the included studies. The individual searches were combined, compared, and reviewed for applicability, where a consensus was made regarding the study inclusion. In case of discrepancies, the review process was repeated and a third researcher was consulted. A K-Cohen’s coefficient of 0.87 indicated a perfect agreement between researchers. Quality assessment was applied independently by the two researchers, using the modified Cochrane methodological quality criteria, and was discussed before the final quality scores assignation (Table 1). The same researchers who screened the titles, abstracts, full texts, and references, also performed the quality assessment. Several domains were identified for categorization of the study results. In particular, studies were analyzed with regards to “anthropometric parameters and body composition”, “cardiovascular parameters and aerobic capacity”, and “blood sample and glucose tolerance”.

## 3. Results

### 3.1. Description of the Study

**Figure 1:**
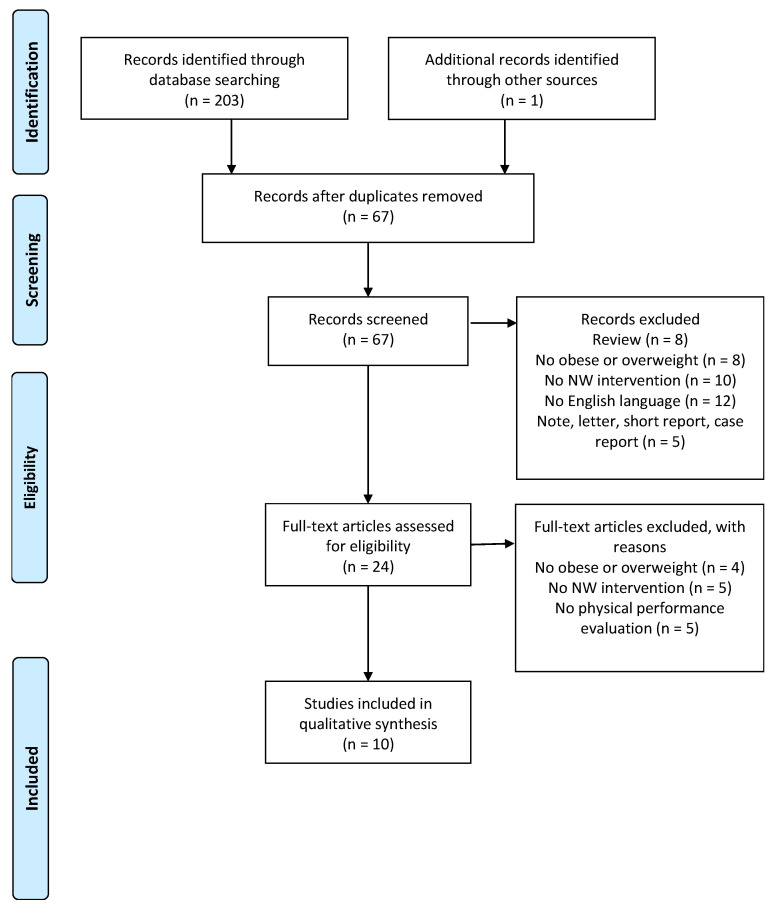
Flow chart.

Two hundred and three studies were found from the literature search. Applying the inclusion and exclusion criteria, 10 were considered eligible for this review (Figure 1). Sample sizes ranged from 17 to 213 subjects, aged from 20 to 80. A single session of NW lasted from 30 to 90 min, and was performed for 1 to 3 times per week (Table 2). The duration of the different study protocols were from 4 to 16 weeks. The intensity of the NW program was reported in 6 studies [19,20,23,24,26,28], and 3 of them provided for the intensity progression [23,24,26]. There was only one case of follow-up, after 32 weeks [28]. Two studies were classified as high quality, while were classified 8 as low [19,20,21,22,24,25,27,28]. Randomization procedures were performed in 5 studies [23,24,25,26,28], and similarity among group was performed in only 1 study [27]. The inclusion and exclusion criteria were adequately reported in 7 studies [19,22,24,25,26,27,28], and only 2 studies described the timing of outcome assessments [20,23]. Blinding procedures were applied in 1 study [23], 7 specified the dropout [19,22,23,24,25,26,28], and 3 performed the intention-to-treat analyses [23,25,28]. Finally, 3 papers reported the compliance [26,27,28]. All results were classified and analyzed with regards to the “anthropometric parameters and body composition” [19,20,21,22,23,24,25,26,27,28], “cardiovascular parameters and aerobic capacity” [19,20,23,24,25,26,27,28], and “blood sample and glucose tolerance” [19,21,22,23,24,25,26,28] (Table 3).

### 3.2. Anthropometric Parameters and Body Composition

All included studies evaluated the anthropometric parameters, and 6 of them recorded significant improvement [19,20,21,22,25,27]. Significant reductions on body weight and BMI were recorded after 4 weeks (body weight −1.3%, BMI −1.3% [19]), 6 weeks (body weight −0.7%, BMI—1.1% [20]), 10 weeks (body weight −5.6%, BMI −5.6% [21]; body weight −6.4%, BMI −6.4% [22]), while after 12 weeks Fabre and colleagues found significant reduction only in body weight (−1.7%) [27]. Fritz and colleagues [25] separately analyzed obese with normal glucose tolerance (NGT), impaired glucose tolerance (IGT), and type 2 diabetes mellitus (T2DM). Sixteen weeks of NW, induced significant reduction of body weight in obese subjects with NGT and T2DM (−2.3%, −1.1%), such as for BMI (−2.4%, −1.3%), while no significant change was found in obese participants with IGT. On the contrary, the authors recorded a significant reduction in waist circumference (WC) in all three groups (NGT −4.9%, IGT −2.2%, and T2DM −1.2%) [25].

Five studies analyzed the body composition [20,23,26,27,28]. Significant reduction in body fat mass (BFM) was recorded after 6 weeks of NW (−0.6%) [20], such as after 12 weeks (−2.2%) [27]. Moreover, Fabre and colleagues found significant reduction in the skin-fold thickness (−7.4%) [27].

### 3.3. Cardiovascular Parameters and Aerobic Capacity

Six studies evaluated the cardiovascular parameters, including the systolic blood pressure (SBP), diastolic blood pressure (DBP), and heart rate (HR) [19,20,25,26,27,28]. The significant changes were, the reduction of HR, at rest, after 6 weeks of NW (−5.9% [20]), and DBP after 12 weeks (−8.1% [27]).

The effects of NW on the aerobic capacity were evaluated in 5 studies [19,23,24,26,28]. After 4 weeks of NW, Kucio and colleagues found a significant improvement in exercise tolerance, expressed as metabolic equivalent values (+17.3%) and time to exhaustion (+14.3%) [19]. After 10 weeks, maximal oxygen consumption (VO_2max_) improved by 17.5% [24].

### 3.4. Blood Sample and Glucose Tolerance

Blood sample was measured and analyzed in 8 studies [19,21,22,23,24,25,26,28]. Low-density lipoprotein (LDL) improved significantly after 10 weeks of NW (−12.2% [21], −16.4% [22]), such as high density lipoprotein (HDL) (+9% [21], +8.1% [22]). Additionally, Fritz and colleagues found a significant difference in HDL between the NW and CG of the NGT group [25]. Total cholesterol (TC) decreased significantly after 4 weeks (−10.3% [19]), and 10 weeks (−9% [21], −12.1% [22]) of NW, while Fritz and colleagues found significant difference between NW and CG in T2DM group [25]. Similarly, triglycerides (TG) decreased after 4 weeks (−33% [19]) and 10 weeks (−21.5% [21], −17.2% [22]) of NW.

Glucose tolerance was analyzed with different methods in 4 studies [23,25,26,28]. In the fasting glucose test, 6 weeks of NW induced a significant decrease in glucose level, monitored for 8-h (−9.6%) [23]. The 2-h oral glucose tolerance test (OGTT-2h) showed a significant improvement after 16 weeks of NW in the IGT (−7.9%) and the T2DM (−11.4%) groups [25]. Homeostasis model assessment of insulin resistance (HOMA-IR) recorded a significant improvement after 6 weeks of NW (−27.8%) [23]. After 6 weeks of NW, the insulin level decreased significantly (−19.3%) [23], and the glycated hemoglobin A1c (HbA1c) decreased after 16 weeks of NW in T2DM group (−4.2%, −6.1%) [25].

## 4. Discussion

This systematic review aimed to analyze the effects of NW programs on overweight and obese subjects. The current results showed positive effects on the anthropometric parameters, body composition, cardiovascular parameters, blood sample, and glucose tolerance. The involvement of the upper and lower limb for performing NW, increased the energy expenditure during the workout, compared to normal walking alone. Moreover, the use of poles reduced the lower limb muscular activation [29], potentially, encouraging individuals with lower exercise tolerance to perform physical activity.

### 4.1. Anthropometric Parameters and Body Composition

Weight control in overweight and obese people was strongly recommended to reduce the risk of developing chronic diseases such as metabolic syndrome and type 2 diabetes. For this reason, lifestyle change, including PA increase and dietary modification was an effective way to improve the overall health. Moreover, weight loss lower than 5% was considered to be not clinically meaningful [30]. According to the previous studies [31,32], a major reduction in body weight and BMI were found in the Derengowska et al. studies, due to the integration of NW and diet control. Moreover, these studies were characterized of a high frequency and duration (60 min 5/6 times per week), with an overall weekly PA of about 300–360 min per weeks [21,22]. A significant reduction in body weight and BMI (5.6% to 6.4%) could be considered as clinically meaningful, confirming the need for a combined PA and diet interventions, for weight loss in overweight and obese people.

From another point of view, even if BMI was found to be the main indicator that helps identify overweight and obese people, WC was a stronger predictor for the risk to develop diabetes than BMI. Indeed, WC was an indicator of body fat distribution, which could identify people with an increased risk for cardio-metabolic disease [33]. Fritz and colleagues analyzed the effect of the non-supervised NW program on the overweight and obese people with NGT, IGT, and T2DM. Despite the long protocol (16 weeks), a significant reduction in body weight and BMI was only recorded in the NGT and T2DM groups. On the contrary, WC was significantly reduced in all the three groups (NGT −4.9%; IGT −2.2%; and T2DM −1.2%). Unfortunately, body fat was not evaluated in this study [25]. Body fat mass were analyzed in three studies that recorded a significant reduction after 12 or 16 weeks of NW (−7.4% [26], −3.4% [28], and −2.2% [27]) despite the minimal weight loss (−2.4% [26], −1.8% [28], and −1.7% [27]). These results highlighted the importance of body composition evaluation, in addition to body weight. In fact, the reduction in total body fat, abdominal obesity or both, without significant weight loss (more than 5%) showed improvements in the cardio-metabolic risk factors, such as insulin sensitivity [34]. Moreover, a major reduction on BFM was recorded in the supervised protocol with intensity progression from 55% to 75% of HR_rest_ [26].

### 4.2. Blood Sample and Glucose Tolerance

Lifestyle change was the primary recommendation for the management of hematic parameters. The lipid profile changes after NW intervention showed contrasting results. Most studies reported improvement in blood lipid profile, but only three investigation displayed a significant reduction of TC, TG, LDL [19,21,22], and only the Derengowska et al. studies found a significant improvement in HDL [21,22]. As described in other research, the integration of diet and PA was more effective on HDL modification [35], and Derengowska and colleagues protocol included both the intervention.

With regards to the metabolic profile, Fritz and colleagues analyzed the effect of 16 weeks of non-supervised NW programs on glucose tolerance and HbAc1 in obese, with or without glucose impairment, such as diabetes mellitus. In their study, participants were required to reach a target of 300 min of non-supervised NW every week. As expected, the results showed a significant reduction in OGTT-2h in obese with IGT and T2DM, while HbAc1 only in T2DM [25]. This protocol was one of the longest and the extensive duration of the NW program, also with the higher adherence (78–96%) and higher minutes of exercise per week, could have concurred to produce these positive results.

In conclusion, exercise frequency and the weekly minutes of non-supervised NW seemed to positively influence the improvement of metabolic control. Results also indicated that a supervised NW program with a shorter duration (6 weeks) could lead to significant improvements in both glucose blood profile and glucose tolerance in middle-aged men with IGT that performed 240 min of activity, with an intensity of 60–75% of HR_max_ [23].

### 4.3. Cardiovascular Parameters and Aerobic Capacity

Aerobic exercise was largely recommended to reduce blood pressure in hypertensive patients. In a recent document, Lopes et al. reported significant reductions in blood pressure, after at least 4 weeks of aerobic exercise, with a different magnitude of improvement among hypertensive, pre-hypertensive, and normal subjects [36]. Despite the protocols of our review being longer than 4 weeks, only one study depicted a significant reduction in DBP [27]. Likewise, these results did not significantly change with a change in heterogeneity of the groups and the hypertensive conditions of the recruited individuals. Despite this, results were aligned with a previous investigation that reported a mean reduction of 3.4 for SBP and 2.4 mmHg of DBP, after aerobic exercise [37]. In fact, the average blood pressure reduction of the included studies reported a 3.4 mmHg reduction for SBP and 2.8 mmHg reduction for DBP. As expected, the aerobic capacity increased after NW intervention. Nevertheless, non-supervised protocol [24] and low weekly minutes of training (only 90 min) [28] reported no significant improvements after NW. On the contrary, it seemed that supervised NW, in which the intensity augmented progressively from 55% to 75% of HR_max_ during the intervention and lasted about 150 to 300 min per week, induced significant improvements of aerobic capacity [19,23,26].

### 4.4. Limitations

This review presented several limitations. First, the included subjects were overweight and obese people, without internal distinction into groups. Second, despite the importance of body fat reduction in people with obesity, body composition was not evaluated in many studies. From this point of view, future investigations should evaluate weight and body composition changes, following a NW protocol, to detect significant clinical changes. Third, diet control was described in only one study, despite it being well-known that the integration of PA and diet is preferable for obesity management. Finally, training modality and intensity were not reported in all the studies and not all included studies specified the prior level of PA in participants, or a minimal level of PA as the inclusion or exclusion criteria for participation. As a consequence, it was difficult to identify a precise dose-response of NW to promote an improvement in each parameter discussed in this systematic review. Moreover, it was not possible to identify a clear and efficient intensity progression to optimize weight loss and improve physical capacity, due to a general lack of information from the analyzed studies.

## 5. Conclusions

The results of this systematic review showed that NW programs could be considered as an effective modality to involve overweight and obese patients in physical activity. Additionally, NW was apparently able to modify different risk factors for cardiovascular diseases, even though the best improvement seemed was observed in a combination of exercise with diet control. To the best of our knowledge, clinicians could consider an NW program as a form of exercise in their prescription to increase PA in overweight and obese people. To promote weight loss, the minimum quantity of recommended NW is 4 times per week and for at least for 60 min per training session. This minimum quantity should preferably be coupled with diet control. 

## Figures and Tables

**Table 1:** Quality assessment of the included studies.

Citation	Randomization Procedure	Similarity of Study Groups	Inclusion or Exclusion Criteria	Dropouts	Blinding	Compliance	Intention-To-Treat Analysis	Timing of Outcomes Assessment	Follow-Up	Results
Kucio et al. (2017) [19]	−	−	+	+	−	−	−	−	−	2/9
Pilch et al. (2017) [20]	−	−	−	−	−	−	−	+	−	1/9
Derengowska et al. (2015) [21]	−	−	−	−	−	−	−	−	−	0/9
Derengowska et al. (2015) [22]	−	−	+	+	−	−	−	−	−	2/9
Wiklund et al. (2014) [23]	+	−	−	+	+	−	+	+	−	5/9
Trabka et al. (2013) [24]	+	−	+	+	−	−	−	−	−	3/9
Fritz et al. (2013) [25]	+	−	+	+	−	−	+	−	−	4/9
Venojärvi et al. (2013) [26]	+	−	+	+	−	+	−	−	−	4/9
Fabre et al. (2011) [27]	−	+	+	−	−	+	−	−	−	3/9
Gram et al. (2010) [28]	+	−	+	+	−	+	−	−	+	5/9

**Table 2:** Characteristics of the studies.

Study	Subjects and Grouping	Training Modality, Program, and Intensity	Duration and Frequency
Kucio et al. (2017) [19]	26 M 47–66 y.o NW (15) CG (11)	Supervised NW Warm up: 10’ Main part: week 1, 30’ of NW at 3 km/h. week 2–4, 40’ of NW at 5 km/h. HR 40–70% of HM_max_	4 weeks 5 d/w 40–50 min
Pilch et al. (2018) [20]	17 F mean 57 y.o. NW (17)	Supervised NW Warm-up: 15’ of walking and dynamic stretching Main part: 60’ of NW on grass and natural surface at 60–70% of HR_max_ Cool-down: 10–15’ of stretching and relaxing exercises	6 weeks 3 d/w 90 min
Derengowska et al. (2015) [21]	32 F 50–68 y.o. NW (32)	Supervised NW Warm-up	10 weeks 5 d/w 60 min
Derengowska et al. (2015) [22]	89 F 50–75 y.o. NW (69) CG (20)	Supervised and no supervised NW Warm-up and cool-down (3 supervised and 3 no supervised)	10 weeks 6 d/w 60 min
Wiklund et al. (2014) [23]	90 F 20–50 y.o. NW (45) DI (45)	Supervised NW Week 1: 60% HR_max_ Week 2–3: 65% HR_max_ Week 4–5: 70% HR_max_ Week 6: 75% HR_max_ DI Reduction of portion size, control meal rhythm, change the composition of food (light margarine, vegetable oil, low-fat milk and meat, vegetables, fruits, increase fiber intake). Drink water, light beverage, mild juice, coffee and tea.	6 weeks 3–4 d/w 30–60 min
Trabka et al. (2013) [24]	46 F NW (25) DI (21)	Supervised NW Warm-up: 10 min Main part: 40 min of NW and 20 min of strength training. HR increased progressively from 50% to 80% of HR_res_, 10% every 2 weeks. Strength exercise was performed in 3 sets. Repetitions were 15 squats, 30 heel-raises, sit-ups until exhaustion, and 15 push-ups on knees. Cool-down: 10 min of stretching DI No high-fat and high-glycemic food Eating 5 times per day, no after 7.00 p.m. or 3 h before sleep Drinking at least 1.5 L of water	10 weeks 3 d/w 80 min
Fritz et al. (2013) [25]	213 (118 F, 95 M) 45–69 y.o. NW-NGT (87) NW-IGT (14) NW-T2DM (20) CG-NGT (75) CG-IGT (21) CG-T2DM (30)	No supervised NW Participants were instructed to increase their weekly level of physical activity by 5 h of NW.	4 months 5 h/w
Venojärvi et al. (2013) [26]	79 M 40–65 y.o. NW (39) CG (40)	Supervised NW Warm-up: walking and stretching of the main muscle group for 5’. Main part: weeks 1–4 at 55% of HR_res_; weeks 5–8 at 65% of HR_res_; weeks 9–12 at 75% of HR_res_. Cool-down: stretching of the main muscle group for 5’.	12 weeks 3 d/w 60 min
Fabre et al. (2011) [27]	23 F NW (12) WT (11)	Supervised and no supervised NW 4 weeks of learning of NW technique before the intervention Warm-up: 5–10’ Main part: 30’ of interval training, 6 bouts of 5 min (4’ at preferred walking speed followed by 1’ at maximal walking speed) Cool-down: 5–10’ 1 supervised and 2 unsupervised NW Supervised and no supervised WT Follow NW but without poles. 1 supervised and 2 unsupervised WT	12 weeks 3 d/w 40–50 min
Gram et al. (2010) [28]	44 (37 M, 31 F) 25–80 y.o. NW (22) CG (22)	Supervised NW Warm-up: 10’ Main part: 30’ (>40% of VO_2max_). Cool-down: 5’	4 months 1–2 d/w 45 min

d/w: day/week; h/w: hour/week; NW: Nordic Walking; CG: Control Group; DI: Diet Group; WT: Walking Training; NGT: Normal Glucose Tolerance; IGT: Impaired Glucose Tolerance; T2DM: Type 2 Diabetes.

**Table 3:** Results of the included studies.

Study	Group Comparison	Results
Kucio et al. (2017) [19]	NW vs. CG	Anthropometric parameters and body composition Weight (kg): ↓NW*; =CG BMI (kg/m^2^): ↓NW*; ↓CG Cardiovascular parameters and aerobic capacity M24-h SBP (mmHg): ↓NW; ↓CG M24-h DBP (mmHg): ↓NW; ↓CG M24-h MAP (mmHg): ↓NW; ↓CG M-daily SBP (mmHg): ↓NW; ↓CG M-daily DBP (mmHg)#: ↓NW; ↓CG M-daily MAP (mmHg): ↓NW; ↓CG M-nightly SBP (mmHg): ↓NW; ↓CG M-nightly DBP (mmHg): ↓NW; ↓CG M-nightly MAP (mmHg): ↓NW; ↓CG Time to exhaustion (min): ↑NW*; ↑CG* MET (mL/kg/min): ↑NW*; ↑CG Blood sample and glucose tolerance TC (mg%): ↓NW*; ↓CG LDL (mg%): ↓NW; ↓CG HDL (mg%): ↓NW; ↓CG TG (mg%): ↓NW*; ↑CG
Pilch et al. (2017) [20]	NW	Anthropometric parameters and body composition Weight (kg): ↓NW* BMI (kg/m^2^): ↓NW* BFM (%): ↓NW* LBM (kg): ↓NW Cardiovascular parameters and aerobic capacity HRrest (bpm): ↓NW*
Derengowska et al. (2015) [21]	NW	Anthropometric parameters and body composition Weight (kg): ↓NW* BMI (kg/m^2^): ↓NW* Blood sample and glucose tolerance TC (mg/dL): ↓NW* LDL (mg/dL): ↓NW* HDL (mg/dL): ↑NW* TG (mg/dL): ↓NW*
Derengowska et al. (2015) [22]	NW vs. CG	Anthropometric parameters and body composition Weight (kg): ↓NW*; ↑CG BMI (kg/m^2^)#: ↓NW*; ↑CG Blood sample and glucose tolerance TC (mg/dL): ↓NW*; ↑CG LDL (mg/dL): ↓NW*,**; ↑CG HDL (mg/dL): ↑NW*,**; ↓CG TG (mg/dL): ↓NW*; ↑CG nHDL (mg/dL): ↓NW*,**; ↑CG Glucose (mg/dL): ↓NW*,**; ↓CG
Wiklund et al. (2014) [23]	NW vs. DI	Anthropometric parameters and body composition Weight (kg)#: ↓NW*; ↓DI* BMI (kg/m^2^)#: ↓NW; ↓DI* BFM (kg)#: ↑NW; ↓DI VFA (cm^2^): ↓NW; ↓DI* FFM (kg): ↓NW; ↓DI* Cardiovascular parameters and aerobic capacity VO_2max_ (mL/kg/min): ↑NW; ↑DI Blood sample and glucose tolerance TC (mmol/L): ↑NW; ↑DI LDL (mmol/L): ↑NW; ↑DI HDL (mmol/L): ↑NW; =DI TG (mmol/L): =NW; =DI Insulin (μU/L): ↓NW*; ↓DI Fasting glucose (mmol/L): ↓NW*,**; ↑DI HOMA-IR: ↓NW*,**; ↓DI
Trabka et al. (2013) [24]	NW vs. DI	Anthropometric parameters and body composition Weight (kg): ↓NW; ↓DI BMI (kg/m^2^): ↓NW; ↓DI WC (cm): ↓NW; ↑DI HC (cm): ↓NW; ↑DI WHR: ↑NW; ↓DI Cardiovascular parameters and aerobic capacity VO_2max_ (mL/kg/min): ↑NW*; ↑DI Blood sample and glucose tolerance TC (mmol/L): ↑NW; ↓DI LDL (mmol/L): ↓NW; =DI* HDL (mmol/L): ↑NW; =DI TG (mmol/L): =NW; =DI
Fritz et al. (2013) [25]	NW vs. CG (NGT)	Anthropometric parameters and body composition Weight (kg): ↓NW*,**; ↓CG BMI (kg/m^2^): ↓NW*,**; ↓CG WC (cm): ↓NW*,**; ↓CG* Cardiovascular parameters and aerobic capacity SBP (mmHg): ↑NW; ↓CG DBP (mmHg): ↑NW; ↓CG Blood sample and glucose tolerance TC (mmol/L): =NW; =CG LDL (mmol/L): =NW; ↑CG HDL (mmol/L): =NW**; ↓CG* TG (mmol/L): =NW; =CG Fasting glucose (mmol/L): =NW; ↓CG* HOMA-IR: ↓NW; =CG OGTT 2h (mmol/L): ↓NW; ↑CG HbA_1c_ (%): =NW; =CG HbA_1c_ (mmol/L): =NW; ↑CG
	NW vs. CG (IGT)	Anthropometric parameters and body composition Weight (kg): ↓NW; ↓CG BMI (kg/m^2^): ↓NW; ↓CG WC (cm): ↓NW*; ↓CG* Cardiovascular parameters and aerobic capacity SBP (mmHg): ↓NW; ↑CG DBP (mmHg): ↑NW; ↓CG Blood sample and glucose tolerance TC (mmol/L): ↑NW; ↓CG LDL (mmol/L): ↑NW; ↓CG HDL (mmol/L): =NW; ↓CG TG (mmol/L): ↓NW; ↓CG Fasting glucose (mmol/L): ↓NW; ↓CG HOMA-IR: ↑NW; ↑CG OGTT 2h (mmol/L): ↓NW*; ↓CG HbA_1c_ (%): ↓NW**; ↑CG HbA_1c_ (mmol/L): ↓NW; ↑CG
	NW vs. CG (T2DM)	Anthropometric parameters and body composition Weight (kg): ↓NW*; ↓CG BMI (kg/m^2^): ↓NW*; ↓CG WC (cm): ↓NW*; ↓CG Cardiovascular parameters and aerobic capacity SBP (mmHg): ↑NW; ↓CG DBP (mmHg): ↓NW; ↓CG Blood sample and glucose tolerance TC (mmol/L): ↓NW**; ↑CG* LDL (mmol/L): =NW; ↑CG* HDL (mmol/L): =NW; =CG TG (mmol/L)#: ↓NW; ↑CG Fasting glucose (mmol/L): ↓NW; ↓CG* HOMA-IR: ↑NW; ↓CG OGTT 2h (mmol/L): ↓NW*; ↓CG* HbA_1c_ (%): ↓NW*; ↓CG HbA_1c_ (mmol/L): ↓NW*; ↓CG
Venojärvi et al. (2013) [26]	NW vs. CG	Anthropometric parameters and body composition Weight (kg): ↓NW**; ↓CG WC (cm): ↓NW; ↓CG BFM (%): ↓NW**; ↓CG FFM (kg): ↑NW; ↓CG Cardiovascular parameters and aerobic capacity SBP (mmHg): ↓NW; ↓CG DBP (mmHg): ↓NW; ↓CG 2-km UKK walk test: ↑NW**; ↑CG Blood sample and glucose tolerance TC (mmol/L): ↓NW; ↑CG LDL (mmol/L): ↓NW; ↑CG HDL (mmol/L): =NW; ↑CG TG (mmol/L): ↓NW; ↓CG Glucose (mmol/L): =NW; ↓CG Insulin (μIU/L)#: ↓NW; ↑CG Insulin 2h (μIU/L)#: ↓NW; ↓CG HOMA-IR#: ↓NW; ↑CG OGTT 2h (mmol/L): ↓NW; ↓CG HbA_1c_ (%): =NW; ↑CG
Fabre et al. (2011) [27]	NW vs. WT	Anthropometric parameters and body composition Weight (kg): ↓NW*; ↓WT* BMI (kg/m^2^): ↓NW; ↓WT BFM (%): ↓NW*; ↓WT* Skin-fold thickness (cm): ↓NW*; ↓WT* Cardiovascular parameters and aerobic capacity SBP (mmHg): ↓NW; ↓WT DBP (mmHg): ↓NW*; ↓WT* HR (bpm): ↑NW; ↑WT
Gram et al. (2010) [28]	NW vs. CG	Anthropometric parameters and body composition Weight (kg): ↓NW; ↓CG BMI (kg/m^2^): ↓NW; ↓CG WC (cm): ↓NW; ↑CG HC (cm)#: ↓NW; ↑CG BFM (kg): ↓NW**; ↑CG FFM (kg): ↓NW; ↓CG Cardiovascular parameters and aerobic capacity SBP (mmHg): ↓NW; ↓CG DBP (mmHg): ↓NW; ↓CG VO_2max_ (L/min): ↑NW; ↑CG Blood sample and glucose tolerance TC (mmol/L): ↑NW; ↓CG LDL (mmol/L): =NW; ↓CG HDL (mmol/L): =NW; =CG TG (mmol/L): ↓NW; ↑CG HbA_1c_ (%): ↓NW; ↑CG

*p* < 0.05, * intra-group difference; ** between group difference; #: significant difference at baseline; ↑: increase; ↓: decrease; =: not change. NW: Nordic Walking; CG: Control Group; DI: Diet Group; NGT: Normal Glucose Tolerance; IGT: Impaired Glucose Tolerance; T2DM: Type 2 Diabetes; WT: Walking Training. T0—baseline; Twn—evaluation after n weeks. BMI—body max index; M-24 h—median 24-h; SBP—systolic blood pressure; DBP—diastolic blood pressure; MAP—mean arterial pressure; M-daily—median daily; M-nightly—median nightly; TC—total cholesterol; LDL—low-density lipoprotein; HDL—high-density lipoprotein; TG—triglycerides; MET—metabolic equivalent; BFM—body fat mass; HRrest—heart rate at rest; nHDL—non-HDL; VFA—visceral fat area; FFM—free fat mass; VO2_max_—maximal oxygen consumption; HOMA-IR—homeostasis model assessment of insulin resistance; WC—waist circumference; HC—hip circumference; WHR—waist-hip ratio; OGTT—oral glucose tolerance test; HbA1c—glycated hemoglobin.

## References

[1] Stamatakis E., Gale J., Bauman A., Ekelund U., Hamer M., Ding D. (2019). Sitting Time, Physical Activity, and Risk of Mortality in Adults. J. Am. Coll. Cardiol..

[2] Ekelund U., Brown W.J., Steene-Johannessen J., Fagerland M.W., Owen N., Powell K.E., Bauman A.E., Lee I.M. (2018). Do the associations of sedentary behaviour with cardiovascular disease mortality and cancer mortality differ by physical activity level? A systematic review and harmonised meta-analysis of data from 850 060 participants. Br. J. Sports Med..

[3] WHO Obesity: Situation and Trends. https://www.who.int/gho/ncd/risk_factors/physical_activity/en/.

[4] Riebe D., Ehrman J.K., Liguori G., Magal M., The American College of Sports Medicine (2018). ACSM’s Guidelines for Exercise Testing and Prescription.

[5] Arem H., Moore S.C., Patel A., Hartge P., Berrington de Gonzalez A., Visvanathan K., Campbell P.T., Freedman M., Weiderpass E., Adami H.O. (2015). Leisure time physical activity and mortality: A detailed pooled analysis of the dose-response relationship. JAMA Intern. Med..

[6] Kokkinos P. (2012). Physical activity, health benefits, and mortality risk. ISRN Cardiol..

[7] Haapanen N., Miilunpalo S., Pasanen M., Oja P., Vuori I. (1997). Association between leisure time physical activity and 10-year body mass change among working-aged men and women. Int. J. Obes. Relat. Metab. Disord..

[8] Di Pietro L., Dziura J., Blair S.N. (2004). Estimated change in physical activity level (PAL) and prediction of 5-year weight change in men: The Aerobics Center Longitudinal Study. Int. J. Obes. Relat. Metab. Disord..

[9] Figard-Fabre H., Fabre N., Leonardi A., Schena F. (2010). Physiological and perceptual responses to Nordic walking in obese middle-aged women in comparison with the normal walk. Eur. J. Appl. Physiol..

[10] Shim J.M., Kwon H.Y., Kim H.R., Kim B.I., Jung J.H. (2013). Comparison of the Effects of Walking with and without Nordic Pole on Upper Extremity and Lower Extremity Muscle Activation. J. Phys. Ther. Sci..

[11] Bullo V., Gobbo S., Vendramin B., Duregon F., Cugusi L., Di Blasio A., Bocalini D.S., Zaccaria M., Bergamin M., Ermolao A. (2018). Nordic Walking Can Be Incorporated in the Exercise Prescription to Increase Aerobic Capacity, Strength, and Quality of Life for Elderly: A Systematic Review and Meta-Analysis. Rejuvenat. Res..

[12] Cugusi L., Solla P., Serpe R., Carzedda T., Piras L., Oggianu M., Gabba S., Di Blasio A., Bergamin M., Cannas A. (2015). Effects of a Nordic Walking program on motor and non-motor symptoms, functional performance and body composition in patients with Parkinson’s disease. NeuroRehabilitation.

[13] Willson J., Torry M.R., Decker M.J., Kernozek T., Steadman J.R. (2001). Effects of walking poles on lower extremity gait mechanics. Med. Sci. Sports Exerc..

[14] Liberati A., Altman D.G., Tetzlaff J., Mulrow C., Gotzsche P.C., Ioannidis J.P., Clarke M., Devereaux P.J., Kleijnen J., Moher D. (2009). The PRISMA statement for reporting systematic reviews and meta-analyses of studies that evaluate health care interventions: Explanation and elaboration. J. Clin. Epidemiol..

[15] Moher D., Liberati A., Tetzlaff J., Altman D.G., Group P. (2009). Preferred reporting items for systematic reviews and meta-analyses: The PRISMA statement. Ann. Intern. Med..

[16] van Tulder M.W., Assendelft W.J., Koes B.W., Bouter L.M. (1997). Method guidelines for systematic reviews in the Cochrane Collaboration Back Review Group for Spinal Disorders. Spine (Phila Pa 1976).

[17] Gobbo S., Bergamin M., Sieverdes J.C., Ermolao A., Zaccaria M. (2014). Effects of exercise on dual-task ability and balance in older adults: A systematic review. Arch. Gerontol. Geriatr..

[18] Vendramin B., Bergamin M., Gobbo S., Cugusi L., Duregon F., Bullo V., Zaccaria M., Neunhaeuserer D., Ermolao A. (2016). Health Benefits of Zumba Fitness Training: A Systematic Review. PM R.

[19] Kucio C., Narloch D., Kucio E., Kurek J. (2017). The application of Nordic walking in the treatment hypertension and obesity. Fam. Med. Prim. Care Rev..

[20] Pilch W., Tyka A., Cebula A., Sliwicka E., Pilaczynska-Szczesniak L., Tyka A. (2017). Effects of a 6-week Nordic walking training on changes in 25(OH)D blood concentration in women aged over 55. J. Sports Med. Phys. Fitness.

[21] Hagner-Derengowska M., Kaluzny K., Hagner W., Kochanski B., Plaskiewicz A., Borkowska A., Bronisz A., Budzynski J. (2015). The influence of a ten-week Nordic walking training-rehabilitation program on the level of lipids in blood in overweight and obese postmenopausal women. J. Phys. Ther. Sci..

[22] Hagner-Derengowska M., Kaluzny K., Kochanski B., Hagner W., Borkowska A., Czamara A., Budzynski J. (2015). Effects of Nordic Walking and Pilates exercise programs on blood glucose and lipid profile in overweight and obese postmenopausal women in an experimental, nonrandomized, open-label, prospective controlled trial. Menopause.

[23] Wiklund P., Alen M., Munukka E., Cheng S.M., Yu B., Pekkala S., Cheng S. (2014). Metabolic response to 6-week aerobic exercise training and dieting in previously sedentary overweight and obese pre-menopausal women: A randomized trial. J. Sport Health Sci..

[24] Trabka B., Zubrzycki I.Z., Ossowski Z., Bojke O., Clarke A., Wiacek M., Latosik E. (2014). Effect of a MAST Exercise Program on Anthropometric Parameters, Physical Fitness, and Serum Lipid Levels in Obese Postmenopausal Women. J. Hum. Kinet..

[25] Fritz T., Caidahl K., Krook A., Lundstrom P., Mashili F., Osler M., Szekeres F.L., Ostenson C.G., Wandell P., Zierath J.R. (2013). Effects of Nordic walking on cardiovascular risk factors in overweight individuals with type 2 diabetes, impaired or normal glucose tolerance. Diabetes Metab. Res. Rev..

[26] Venojarvi M., Wasenius N., Manderoos S., Heinonen O.J., Hernelahti M., Lindholm H., Surakka J., Lindstrom J., Aunola S., Atalay M. (2013). Nordic walking decreased circulating chemerin and leptin concentrations in middle-aged men with impaired glucose regulation. Ann. Med..

[27] Figard-Fabre H., Fabre N., Leonardi A., Schena F. (2011). Efficacy of Nordic walking in obesity management. Int. J. Sports Med..

[28] Gram B., Christensen R., Christiansen C., Gram J. (2010). Effects of nordic walking and exercise in type 2 diabetes mellitus: A randomized controlled trial. Clin. J. Sport Med..

[29] Sugiyama K., Kawamura M., Tomita H., Katamoto S. (2013). Oxygen uptake, heart rate, perceived exertion, and integrated electromyogram of the lower and upper extremities during level and Nordic walking on a treadmill. J. Physiol. Anthropol..

[30] Bouchard C., Blair S.N., Haskell W. (2012). Physical Activity and Health.

[31] Foster-Schubert K.E., Alfano C.M., Duggan C.R., Xiao L., Campbell K.L., Kong A., Bain C.E., Wang C.Y., Blackburn G.L., McTiernan A. (2012). Effect of diet and exercise, alone or combined, on weight and body composition in overweight-to-obese postmenopausal women. Obesity (Silver Spring).

[32] Swift D.L., Johannsen N.M., Lavie C.J., Earnest C.P., Church T.S. (2014). The role of exercise and physical activity in weight loss and maintenance. Prog. Cardiovasc. Dis..

[33] Klein S., Allison D.B., Heymsfield S.B., Kelley D.E., Leibel R.L., Nonas C., Kahn R., Association for Weight M., Obesity P., Naaso T.O.S. (2007). Waist circumference and cardiometabolic risk: A consensus statement from Shaping America’s Health: Association for Weight Management and Obesity Prevention; NAASO, The Obesity Society; the American Society for Nutrition; and the American Diabetes Association. Am. J. Clin. Nutr..

[34] Ross R., Janssen I., Dawson J., Kungl A.M., Kuk J.L., Wong S.L., Nguyen-Duy T.B., Lee S., Kilpatrick K., Hudson R. (2004). Exercise-induced reduction in obesity and insulin resistance in women: A randomized controlled trial. Obes. Res..

[35] Bouaziz W., Schmitt E., Kaltenbach G., Geny B., Vogel T. (2015). Health benefits of endurance training alone or combined with diet for obese patients over 60: A review. Int. J. Clin. Pract..

[36] Lopes S., Mesquita-Bastos J., Alves A.J., Ribeiro F. (2018). Exercise as a tool for hypertension and resistant hypertension management: Current insights. Integr. Blood Press Control..

[37] Fagard R.H. (2001). Exercise characteristics and the blood pressure response to dynamic physical training. Med. Sci. Sports Exerc..

